# Patients’ perspectives on the effect of the COVID-19 pandemic on access to cancer care and social contacts in Sweden and the UK: a cross-sectional study

**DOI:** 10.1007/s00520-022-07298-7

**Published:** 2022-08-19

**Authors:** Karolina Edlund, Lisen Arnheim Dahlström, Anna Mia Ekström, Mia L. van der Kop

**Affiliations:** 1 Department of Anthropology, Kenyon College, Gambier, OH USA; 2 War On Cancer, Stockholm, Sweden; 3 Department of Global Public Health, Karolinska Institutet, 171 77 Stockholm, Sweden; 4 Department of Infectious Diseases, South Central Hospital, Stockholm, Sweden

**Keywords:** Cancer patient, Coronavirus disease, UK, Sweden, Social isolation, Healthcare access

## Abstract

**Purpose:**

We aimed to determine whether there was a difference in access to cancer-related healthcare between people living in Sweden and the United Kingdom (UK) during the COVID-19 pandemic. We also describe how the pandemic affected social contact of patients undergoing treatment.

**Methods:**

This cross-sectional study used survey data collected through the War on Cancer mobile phone application between September 5, 2020, and January 6, 2021. We included individuals with cancer diagnoses living in Sweden or the UK. The association between difficulty accessing cancer-related healthcare and country was examined using logistic regression. Frequencies were used to describe the effect of the pandemic on social contact.

**Results:**

Of 491 individuals included in the study, 183 were living in the UK and 308 in Sweden. Living in the UK was associated with greater difficulty accessing cancer-related healthcare (*n* = 99/183, 54.1%) than living in Sweden (*n* = 100/308, 32.5%) (odds ratio 2.12, 95% CI 1.39–3.23, *p* < 0.001). The pandemic affected social contact for almost all patients (*n* = 218/238, 91.6%) undergoing treatment.

**Conclusion:**

This study highlights the differential impact that the pandemic may have had on patients’ access to cancer-related care in the UK and Sweden. In both countries, the pandemic overwhelmingly affected social contact of individuals undergoing cancer treatment. New ways must be found to improve access to cancer-related care and reduce social isolation for patients with cancer during a pandemic.

## Introduction 

The COVID-19 pandemic has challenged healthcare systems in unprecedented ways. By putting pressure on medical resources and isolating us from one another, the coronavirus has indirectly affected people with other illnesses, including those living with cancer. In 2018, over 17 million people were diagnosed with cancer, and one in roughly two people living in high-income countries are expected to be diagnosed with the disease within their lifetime [1, 2]. Patients with cancer face additional stressors during the pandemic such as delays and interruptions in treatment schedules and depression due to social isolation [3]. Reports from oncologists across Europe indicate that access to cancer-related care has been affected, and models predict a surge in delayed cancer diagnoses and reduced cancer survival in the aftermath of the pandemic [4–6]. Despite these unique effects of the pandemic on access to care and cancer outcomes, the effects of the COVID-19 crisis from the perspective of those living with cancer remain largely unknown.

National responses to COVID-19 have varied along a continuum, and it is important to consider the regulatory context when characterizing experiences for those living with cancer in different countries. This is particularly true when comparing Sweden and the UK, two countries with similar healthcare systems but that took markedly different approaches to managing the pandemic. Beginning in the fall of 2020, the Swedish government prohibited large-scale public gatherings, asked citizens to refrain from gathering in private homes with more than eight people, and recommended that adults and many students work and study remotely. With these predominantly voluntary measures, Sweden did not go into national “lockdown”. Rather, the Swedish government maintained their belief in citizens’ collectivist values and had a more “open” approach to the COVID-19 pandemic. In contrast, in November 2020, the UK instated a national lockdown, which prohibited gathering indoors. Those living alone were exempt from this mandate and were allowed a support “bubble” linking two households. Towards the end of this study in December 2020, many regions of the UK were once again allowed gatherings of up to six people. The UK’s response represents a more legislatively stringent approach to the pandemic than Sweden’s approach, as the UK’s measures were mandatory rather than voluntary.

In both scenarios, face-to-face data collection for public health purposes became more difficult. Mobile phone applications (apps) are increasingly being used to collect survey data because they do not require physical contact and cover large geographic areas quickly and at low cost [7]. This study used the War on Cancer app, which aims to support those living with cancer. We administered a survey through the app to explore and compare perceptions of how the pandemic affected access to cancer-related care and social contacts from the perspective of individuals living with cancer in Sweden and the UK.

### Objectives

1. Determine whether there was a difference in patient perspectives of their access to cancer-related healthcare between those living in Sweden and those living in the UK during the pandemic.
2. Identify the types of difficulties that participants had accessing cancer-related care.
3. Describe the effect of the pandemic on social contacts for those undergoing cancer treatment.
4. Determine whether employers adapted to the needs of patients who considered themselves at high risk of contracting or getting seriously ill from COVID-19.

## Methods

### Study design

This cross-sectional study used survey data collected through the mobile phone app “War on Cancer” (waroncancer.com). This app, launched in 2016, aims to establish a digital community where people share and connect with others also experiencing cancer. During the study period, War on Cancer had just under 6000 members from across the globe, including those with cancer and loved ones of those with cancer. Most members are female and between the ages of 30 and 50. The majority are living in Sweden and the UK. Content is available in two languages, Swedish and English, although it may be translated to additional languages within the app. The platform is free of charge. Once registered, members can post their own stories, react and respond to others’ stories, send messages in private groups, and take part in webinars, podcasts, and health surveys. Members can also search for relevant clinical trials.

This study included those who had been diagnosed with cancer living in Sweden or the UK during part of the first and second waves of the COVID-19 pandemic (defined as being before or after November 15, 2020, respectively). Data were collected from September 5, 2020, to January 5, 2021. A team of researchers from the UK and Sweden, including epidemiologists and cancer care clinicians, formulated the survey. Questions focused on how the COVID-19 pandemic affected participants’: (1) access to cancer-related healthcare, (2) access to social contacts for those undergoing cancer treatment, and (3) employer support for those who were employed.

**Fig. 1:**
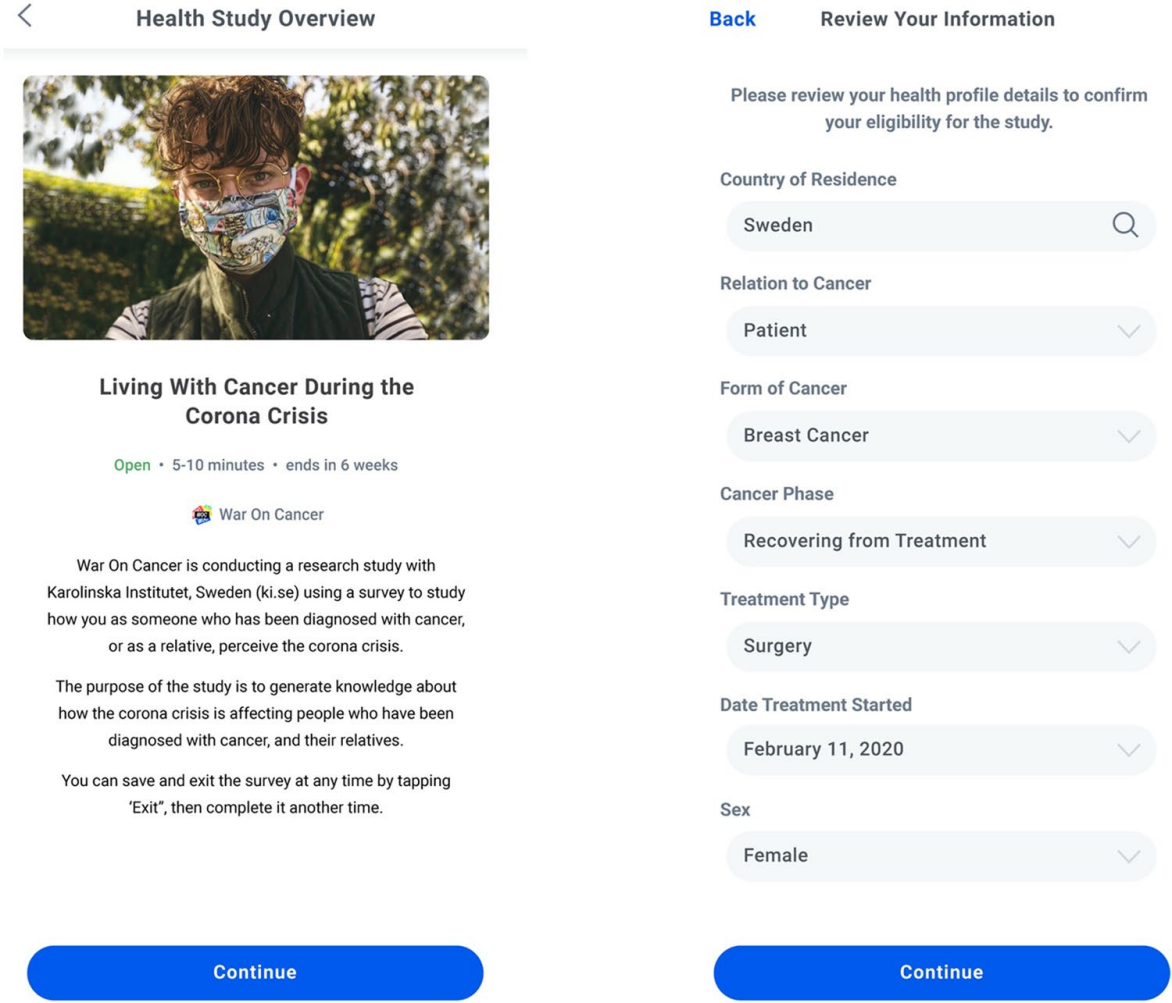
War on Cancer in-app survey interface

All app users were welcome to complete the survey (Fig. 1), but only those who indicated that they were living in the UK or Sweden were included in this study. Participants who were loved ones of those with cancer were excluded, as were those who completed fewer than eight of the 12–14 survey questions. There were no exclusion criteria with respect to age, gender, or cancer type. All participants provided informed consent before completing the questionnaire.

The primary outcome was reported difficulty accessing cancer-related healthcare. The type of difficulty was identified, including difficulties getting questions answered; postponed examinations (e.g., blood samples, specialist visits); postponed treatments (e.g., chemotherapy, radiation); and other types of difficulties. Responses to these questions were coded 0 (no) or 1 (yes). Multiple responses were permitted, but any one indication of a difficulty was considered as having a difficulty accessing cancer-related healthcare.

Whether the pandemic influenced visits from social contacts for those undergoing cancer treatment were also coded 0 (no) or 1 (yes). Participants had the opportunity to select multiple ways in which the pandemic affected their contact socially. Similarly, for those who were employed and considered themselves at high risk of contracting COVID-19 or becoming seriously ill from the virus, whether their employer met their needs was coded as 0 (no) and 1 (yes). Demographic data, such as age, country of residence, gender, and type of cancer, were also collected.

The number of participants who responded to the survey during the study timeframe determined sample size. It is accepted that logistic regression models should have a minimum of 10 outcome events per variable included in the model. With 199 events of difficulties accessing cancer-related healthcare in this cohort, our sample size was adequate to build a stable model with the four selected variables [8].

### Statistical analyses

Descriptive analyses of the study population were conducted using frequencies for categorical variables and mean and standard deviation (SD) for continuous variables. Histograms and measures of skewness and kurtosis were used to assess normality of continuous variables before summarizing the data using means. For the primary analysis, a univariable analysis was used to examine the crude association between country and difficulty accessing cancer-related healthcare. Then the following potentially confounding variables were considered: age (≤ 45 years v. > 45 years); sex (male v. female); wave of the pandemic (first wave v. second wave); and type of cancer (breast, cervical, lung, other). These variables were included in an initial logistic regression multivariable model and retained in the final model if their *p*-value was ≤ 0.25. Odds ratios (OR) alongside 95% confidence intervals and *p*-values are reported. A two-sided *p*-value of less than 0.05 was considered significant. We performed descriptive analyses for secondary outcomes and reported the results as frequencies and proportions. Descriptive analyses were conducted in SPSS 28 (IBM Corp. Released 2020. IBM SPSS Statistics for Windows, version 27.0. Armonk, NY: IBM Corp), and the logistic regression model was built in Stata 12 (StataCorp. 2011. Stata Statistical Software: Release 12. College Station, TX: StataCorp LP).

## Results

**Fig. 2:**
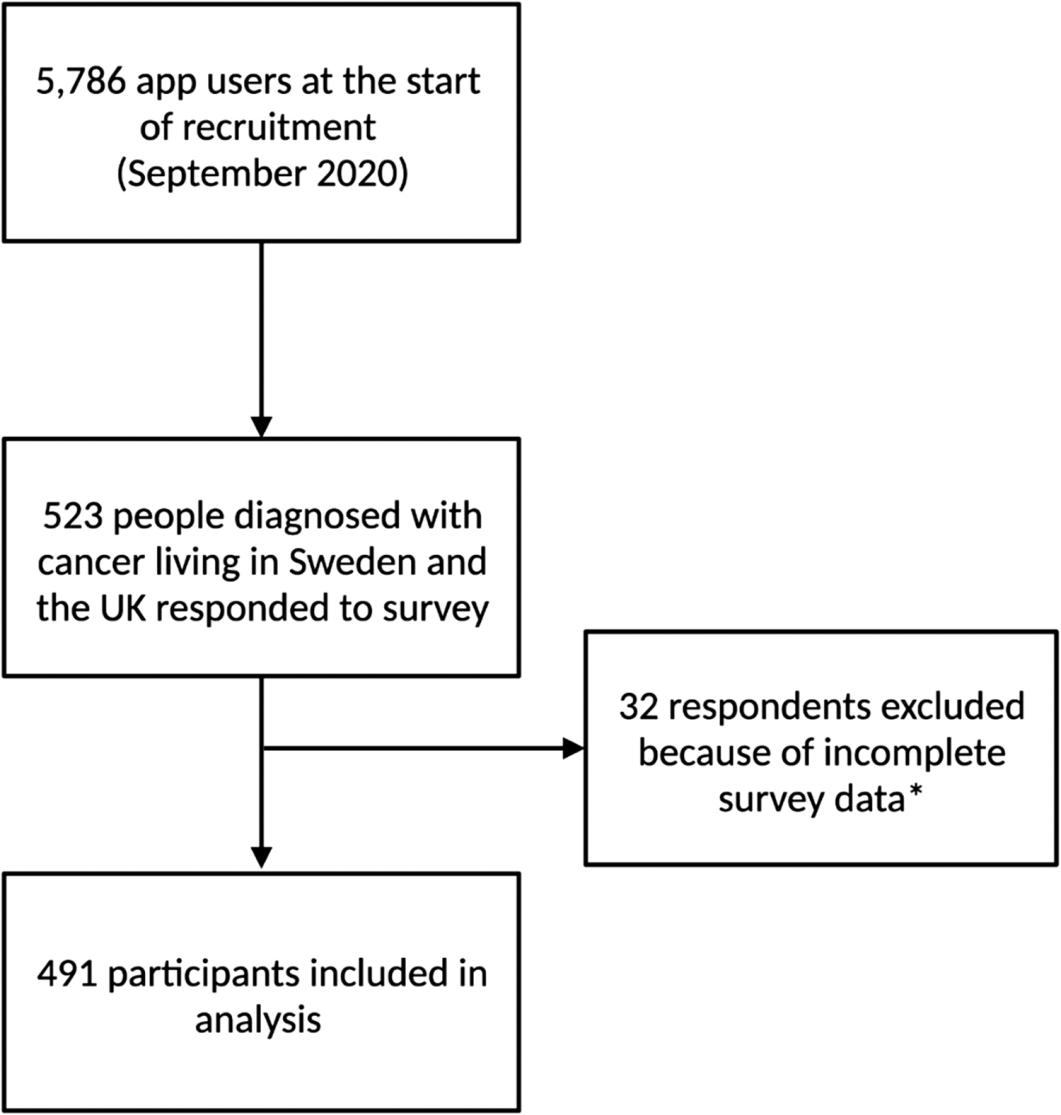
Participant flow chart. Incomplete responses were defined as those with less than 8 of the 12–14 survey questions answered

Between September 5, 2020, and January 6, 2021, 523 people living in Sweden or the UK who identified as having been diagnosed with cancer completed the survey, 491 of whom were included in the study (Fig. 2). More participants were from Sweden (62.7%) than the UK (37.3%), and the mean age of respondents was 44 years old (SD 10.93). Most participants were female (84.7%). Additionally, 56.8% of responses were submitted during the first wave of the pandemic, while 43.2% were submitted during the second wave (Table 1).

**Table 1:** Baseline characteristics

	Sweden (*n* = 308)	UK (*n* = 183)
*Gender*		
Female	266 (86.36%)	150 (81.97%)
Male	41 (13.31%)	33 (18.03%)
Other/rather not say	1 (0.32%)	0 (0.00%)
*Age*		
Mean (standard deviation)	44.41 (11.72)	43.83 (9.48)
≤ 45	158 (51.30%)	92 (50.27%)
> 45	150 (48.70%)	91 (49.73%)
*Cancer diagnosis*		
Breast	110 (35.71%)	82 (44.81%)
Cervical	16 (5.19%)	12 (6.56%)
Lung	12 (3.90%)	9 (4.92%)
Other	161 (52.27%)	77 (42.08%)
Missing data	9 (2.92%)	3 (1.64%)
*COVID-19 pandemic wave*		
First	125 (40.58%)	154 (84.15%)
Second	183 (59.42%)	29 (15.85%)
*COVID-19 test*		
Positive test	22 (7.14%)	8 (4.37%)
Negative test	164 (53.25%)	113 (61.75%)
No test	117 (37.99%)	61 (33.33%)
Missing data	5 (1.62%)	1 (0.55%)
*Treatment*		
Undergoing treatment*	141 (45.78%)	97 (53.01%)
Not undergoing treatment	159 (51.62%)	80 (43.72%)
Missing data	8 (2.60%)	6 (3.28%)
*Employment status*		
Employed	230 (74.68%)	113 (61.75%)
Not employed	67 (21.75%)	63 (34.43%)
Missing data	11 (3.57%)	7 (3.83%)

^*^Of the 141 participants undergoing treatment in Sweden, 21 (14.89%) reported doing so exclusively at the hospital, 93 (65.96%) exclusively at home, and 27 (19.15%) at both. Of the 97 participants undergoing treatment in the UK, 16 (16.49%) reported doing so exclusively at the hospital, 52 (53.61%) exclusively at home, and 29 (29.90%) at both

**Fig. 3:**
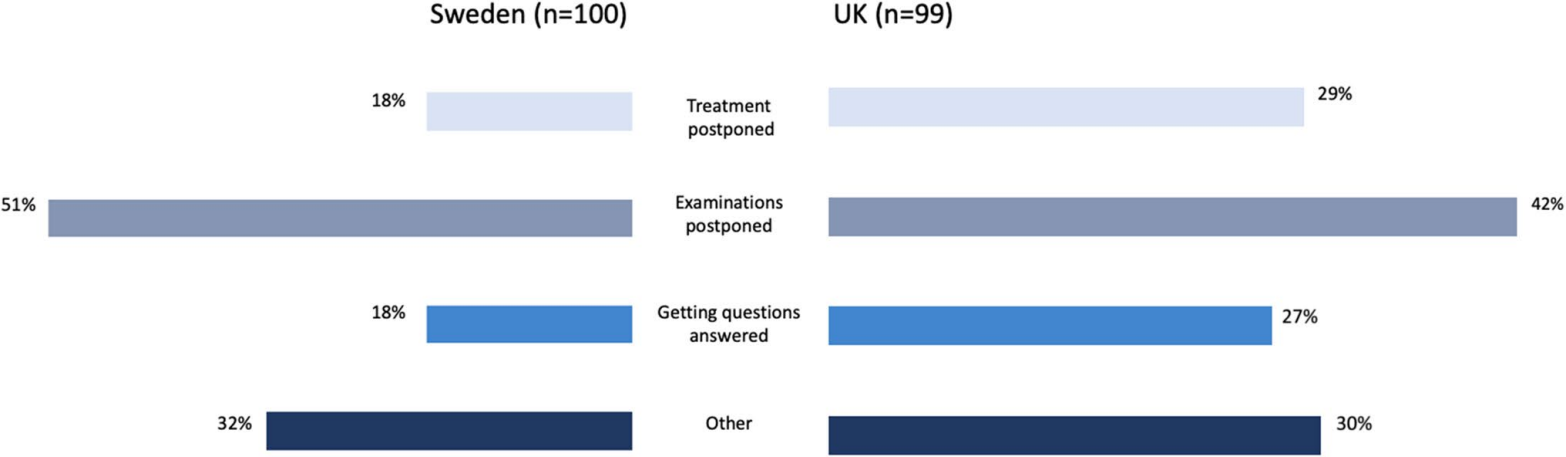
Type of difficulty accessing cancer-related healthcare^*^ by country. *Answers are not mutually exclusive

Those living in the UK had more difficulties accessing cancer-related healthcare than those in Sweden. In the UK, 54.7% of respondents had difficulties compared to 33.3% respondents living in Sweden. Data were missing for 10 participants. Patients in the UK were more likely to have had difficulty accessing cancer-related care than those in Sweden (crude odds ratio 2.41, 95% confidence interval 1.65–3.53, *p* < 0.001) (Table 2). After adjustment for age and wave of the pandemic, this finding remained significant (adjusted odds ratio 2.12, 95% CI: 1.39–3.23; *p* < 0.001). Sex and type of cancer did not significantly contribute to the model. The primary difficulty participants had accessing cancer-related healthcare in both countries was their examinations being postponed (51.0% in Sweden and 42.4% in the UK) (Fig. 3).

**Table 2:** Multivariable logistic regression results for country and difficulty accessing cancer-related care during the COVID-19 pandemic

Factor	Crude OR (95% CI), *p*-value	Adjusted^*^ model: OR (95% CI), *p*-value	Final model^†^: OR (95% CI), *p*-value
UK vs Sweden	2.41 (1.65–3.53), < 0.001	2.19 (1.42–3.36), < 0.001	2.12 (1.39–3.23), < 0.001

^*^Adjusted for gender, age, pandemic wave, and cancer diagnosis

^†^Adjusted for age and pandemic wave

Next, we looked at the pandemic affected participants’ access to their social contacts while undergoing treatment. Of those included in the study, 238 were receiving cancer treatment: 201 at hospital, 93 at home, and 56 both at the hospital and at home. The pandemic affected 91.6% of participants’ access to social contacts, with 92.2% (*n* = 130/141) and 90.7% (*n* = 88/97) of respondents reporting that their social contact was affected in Sweden and the UK, respectively. Fear appeared to play a larger role in people’s access to social contacts for those being treated at home than it did for those being treated in hospital, with 33.8% (*n* = 68/201) of home-treated patients compared to 8.6% (*n* = 8/93) of hospital-treated patients reporting fear of contracting the virus from visitors (Table 3).

**Table 3:** Access to social contacts by treatment location and country

	Sweden	UK
*Overall*	**(*n* = 141)**	**(*n* = 97)**
Social contact not affected	11 (7.8%)	9 (9.3%)
Social contact affected	130 (92.2%)	88 (90.7%)
*Treated at hospital*	**(*n* = 48)**	**(*n* = 45)**
Visitors are allowed	2 (4.2%)	4 (8.9%)
Only a few visitors are allowed	7 (14.6%)	9 (20.0%)
No visitors are allowed	33 (68.8%)	26 (57.8%)
Fear of contracting the virus from visitors	3 (6.3%)	5 (11.1%)
Visitors fearful of transmitting the virus to the participant	7 (14.6%)	12 (26.7%)
Other	5 (10.4%)	1 (2.2%)
*Treated at home*	**(*n* = 120)**	**(*n* = 81)**
People are allowed to visit as before	13 (10.8%)	16 (19.8%)
Only allow a few visitors	71 (59.2%)	41 (50.6%)
Doesn’t allow visitors because of fear of contracting the virus	40 (33.3%)	28 (34.6%)
Fear transmitting the virus to visitors	2 (1.7%)	4 (4.9%)

Of those who were employed and considered themselves at high-risk (*n* = 274/491, 55.8%), 89.8% (*n* = 246/274) of individuals felt that their employer catered to their needs. The proportion who felt that their employer was supportive was similar in both settings, with 91.0% of respondents in Sweden and 87.6% of respondents in the UK agreeing with this statement. The question on employment was not applicable to 40.5% (*n* = 199/491) of the respondents because they were either not employed or did not consider themselves high risk of contracting COVID-19 or getting seriously ill from the virus. Additionally, data were missing for 18/491 (3.7%) participants.

## Discussion

In this study, respondents from the UK experienced more difficulties accessing cancer-related healthcare than their Swedish counterparts. The greatest difficulty reported by participants in both countries was having their examinations postponed. In Sweden and the UK, the pandemic overwhelmingly affected participants’ access to their social contacts, regardless of where participants received treatment. Most employed respondents in both nations felt that their employer catered to their needs.

While Sweden and the UK’s healthcare systems differ, they are comparable in some ways. Both nations have universal healthcare systems and face similar population-level health challenges, such as an aging population and high rates of cancer. In response to the pandemic, both nations’ healthcare systems reprioritized resources in attempt to curb the spread of the virus and to provide care for those who became ill with COVID-19. Such adjustments impacted all medical services, including those receiving care along the cancer care continuum, comprised of screening, early detection, diagnosis, treatment, survivorship, and end-of-life care [9]. Numerous high-income countries in Europe, including Sweden and the UK, have reported decreased access to other medical and emotional resources during the pandemic [10]. As such, the potential for a post-pandemic surge in reduced cancer survival appears likely [11]. This study adds a patient perspective to these reports, with patients from both countries having difficulties accessing cancer-related care, primarily related to having their examinations postponed [12, 13]. These findings are supported by a systematic review indicating that people who have cancer experienced significant delays in their follow-up appointments, treatment, and lack of access to other aspects of cancer care during the COVID-19 pandemic [14]. In this study, differences in access to cancer-related care between Sweden and the UK during the pandemic may be related to pre-pandemic differences in cancer care access between the two countries. Research suggests that cancer care in the UK lags behind that in Sweden, and that late diagnoses, delayed access to treatment, and age bias are likely factors for England’s poor cancer survival rates [15, 16]. Our results suggest that this gap in access to cancer care between these two countries persisted through the COVID-19 pandemic.

More than 90% of respondents in Sweden and the UK reported that the pandemic affected their access to social contacts, which is concerning, as previous research has linked social connectedness with improved cancer survival [17]. In 2019, Braun et al. found that frequent contact with family, friends, and religious communities decreased the risk of death from any cause by 15–28% [18]. Other populations in Europe, including those surveyed using the European Organization for Research and Treatment of Cancer quality of life questionnaire, also experienced a significant decline in social functioning and social isolation during the pandemic [19, 20]. Despite the differences in Sweden’s and the UK’s approaches to managing social gatherings during the pandemic, patient reports of reduced access to social support appear similar between the two countries. This finding may suggest that in this case, access to social contacts is mediated more by individuals themselves than by national regulation. As access to social contacts did not differ much between Sweden and the UK, it appears that voluntary recommendations were enough to establish social norms of social distancing in Sweden. Nonetheless, the problem of finding ways to safely maintain access to social support during apandemic needs to be addressed, particularly as a lack of social support is a risk factor for loneliness [21]. Clinicians may help alleviate the detrimental impact of the pandemic on social support by informing patients of new ways to ensure continued support. This includes informational support through telehealth programs, on-line peer support and group psychosocial support programs, and encouragement to maintain the support of family and friends via telephone and on-line [22, 23].

For many cancer survivors, work plays a key role in maintaining feelings of normalcy, control, and financial security. Working, and feeling supported at work, has been shown to improve quality of life for cancer survivors and those around them [24]. Because the immunosuppressed status of some patients living with cancer puts them at greater risk of developing serious complications if they were to become infected with COVID-19, it may be of particular benefit for this population to work remotely for their health and safety, even after other employees return to work in-person [25]. Other studies have found that people with cancer experienced job loss or a reduction of hours during the pandemic [26], and that they were less likely to work from home than people without cancer and other disabilities [27]. In our study, most participants in both Sweden and the UK felt that their employers catered to their needs, which is encouraging. Maintaining this type of support for those living with cancer during the ongoing COVID-19 crisis and future pandemics is critical to their well-being and financial stability [28].

This study has several strengths. First, while there are many comparative studies of cancer treatment and outcomes between countries, and studies of single-country experiences of those with cancer during the COVID-19 pandemic, ours is the first to contrast the experiences of those living with cancer in two countries during the pandemic [29, 30]. Second, the two countries compared in the present study have important similarities and differences — both Sweden and the UK are classified as high income and have national healthcare systems, yet they differed markedly in their national response strategies to COVID-19. This study highlights the usefulness of mobile applications, such as the War on Cancer app, as a means of data collection during times of decreased social contact. Limitations of this study include the potential for volunteer bias. Those who subscribe to the War on Cancer app may have had different experiences than the general population of those with cancer living in Sweden or the UK, which may have affected their experiences accessing cancer-related healthcare or changes to their social contacts during the pandemic. Furthermore, while we were able to adjust our analyses for confounders such as age and wave of the pandemic, we were unable to control for other potentially important confounding factors such as ethnicity and education.

Our comparison between patients with cancer living in Sweden and the UK allows us to better understand how the COVID-19 pandemic impacted their perspectives on access to cancer care, social contact, and employer support. Like people with cancer, those who have a chronic disease require regular disease management and follow-up, which was likely affected by the pandemic. Social contact with others outside of their household also provides benefits to those with chronic diseases compared to the general population [31]. Our findings may be generalizable to those with chronic disease, although research specific to these populations may be more informative. Further research is required to understand the unique fears and worries of patients with cancer during the pandemic as well as the long-term impacts of treatment delays and reduced social contact on their mental health and clinical outcomes. This study emphasizes the importance of finding new ways to ensure that individuals living with cancer are supported during the ongoing COVID-19 pandemic and future public health crises.

## Data Availability

Not applicable.
